# Characterization of Humic Substances in the Soils of *Ophiocordyceps sinensis* Habitats in the Sejila Mountain, Tibet: Implication for the Food Source of *Thitarodes* Larvae

**DOI:** 10.3390/molecules24020246

**Published:** 2019-01-10

**Authors:** Yan Li, Lian-Xian Guo, Qian-Zhi Zhou, Di Chen, Jin-Zhong Liu, Xiao-Ming Xu, Jiang-Hai Wang

**Affiliations:** 1 Guangdong Provincial Key Laboratory of Marine Resources and Coastal Engineering, School of Marine Sciences, Sun Yat-Sen University, Guangzhou 510006, China; liyan255@mail.sysu.edu.cn (Y.L.); zhouqzhi@mail2.sysu.edu.cn (Q.-Z.Z.); 2 Southern Laboratory of Ocean Science and Engineering (Guangdong, Zhuhai), Zhuhai 519000, China; 3 Dongguan Key Laboratory of Environmental Medicine, School of Public Health, Guangdong Medical University, Dongguan 523808, China; glx525@163.com; 4 South China Sea Institute of Oceanology, Chinese Academy of Sciences, Guangzhou 510301, China; cd0304@163.com; 5 State Key Laboratory of Organic Geochemistry, Guangzhou Institute of Geochemistry, Chinese Academy of Sciences, Guangzhou 510640, China; liujinz@gig.ac.cn

**Keywords:** humic substance, molecular structure, nutritional evaluation, lipid, *Thitarodes* larva

## Abstract

Humic substances in soil are considered to be an alternative food to the tender plant roots for *Thitarodes* larvae in the habitats of *Ophiocordyceps sinensis* in the Qinghai-Tibetan Plateau. However, there is no report involving the evaluation of their potential as a food source from the composition and structure of habitat soils. In this work, the composition and structure of humic substances in habitat soils from the Sejila Mountain, Tibet were characterized by diverse techniques for evaluating the nutritional value and possibility of humus as the food source for *Thitarodes* larvae. Fourier transform infrared spectroscopy revealed that humic acid may possess superior ability to provide the molecular segments for biosynthesizing lipids more than other humic fractions. Combining with the analysis of solid-state ^13^C nuclear magnetic resonance spectrum, the fractions of hydrophobic fulvic acid and hydrophilic fulvic acid are further considered as a potential food source for *Thitarodes* larvae. Overall, humic substances in habitat soils are rich in the molecular segments for biosynthesizing lipids and other important nutrients, which may provide the energy and material sources for maintaining the survival of *Thitarodes* larvae in the absence of tender plant roots, particularly in the annual cold winter. Combining with the evidence of physico-chemical parameters of habitat soils and stable carbon isotopic composition of major tender plant roots in the Sejila Mountain, the composition and structure of humic substances in habitat soils may provide a novel idea for the eco-friendly and semi-wild cultivation of *Thitarodes* larvae with low cost.

## 1. Introduction

*Ophiocordyceps sinensis* (*O. sinensis*) is a precious medicinal fungus natively produced in the Qinghai-Tibetan Plateau and its adjacent high-altitude area [1,2,3]. Owing to its unique medicinal/health care efficacy [4,5], *O. sinensis* has been tremendously popular as a rare health food in China for more than 2000 years [6]. The pronounced beneficial effects have resulted in a large demand for wild *O. sinensis*. However, the wild resource is extremely scarce due to its obligate parasitism and special dependence on ecological environments [2,3,7,8]. Worse still, damages to the habitat ecosystem caused by human activities just as overexploitation and overgrazing further lead to a continuous decline in its yield [9].

To alleviate the tension between the ecological environment and demand for natural *O. sinensis*, improving its artificial cultivation and developing its alternative substitutes have already become hot topics [10]. However, the supply of wild *O. sinensis* cannot fully meet the pharmaceutical and health-care demands, due to the great difference in the medicinal efficacy between wild *O. sinensis* and its substitutes [11]. The semi-natural cultivation of *O. sinensis* has been investigated for many years [9], but its massive fosterage with low cost is still unresolved. Obviously, to achieve this goal, it is necessary to solve the following important problem, that is, how to enhance the yield of *O. sinensis* via the artificial intervention of synchronously improving the density of *Hepialus* larvae and their infection rate by *O. sinensis* in natural habitats [6,12].

The diet of *Thitarodes* larvae in the Qinghai-Tibetan Plateau has been systematically studied with conventional methods, and the results suggest that the larvae are omnivorous, preferring to eat the tender roots of Polygonaceae plants [13,14,15,16,17,18]. Our group investigated the diet of *Thitarodes* larvae in the Sejila Mountain, Tibet by stable carbon isotope analysis, and revealed for the first time that humic substances in habitat soils might be their alternative food source [12]. This discovery provides a novel clue for the eco-friendly cultivation of *Thitarodes* larvae with low cost. Clearly, it is necessary to evaluate the nutritional value of habitat soils and discuss the possibility of humic substances as an alternative food source for *Thitarodes* larvae.

Humic substances in soils have been thoroughly studied, particularly the relationship between their chemical structures and soil fertility [19,20,21,22,23]. Their beneficial effects on plants have been widely investigated [24,25], indicating that they may promote the utilization and maintenance of nutrients in soils. The mechanism of enhancing soil fertility has been further expounded, suggesting that humic substances may drive many physical, chemical and biological processes for improving the physico-chemical properties of soils and bioavailability of nutrients in soils [21,26].

Recent studies have developed the concepts of molecular and supramolecular structures of humic substances [27,28]. Their novelty is that the molecular masses of humic substances are determined to be much lower than those reported earlier [29]. Due to the structural complexity and spatial heterogeneity of humic substances in habitat soils, multiple analytical methods have to be employed just for measuring their elemental composition and functional groups, which are closely related to biosynthesis of nutritive substances, especially fats. It has been demonstrated that the techniques of Fourier transform infrared spectroscopy (FTIR) and solid-state ^13^C nuclear magnetic resonance spectrum (^13^C NMR) are two powerful tools for investigating complicated compounds at the molecular level [22,30,31,32,33]. It is consensus that solid-state ^13^C NMR spectroscopy is not an acceptable method to quantitatively determine soil organic matters, and may even lead to incorrect conclusions, such as the statement that humic acids are predominantly aliphatic [29]. Despite of the existence of the criticisms on the quantitative use of ^13^C NMR spectroscopy, this spectroscopic method is still adopted to qualitatively study soil organic matters. In comparison with the other methods, such as chemical and thermal degradation, the two abovementioned methods are non-destructive, and may provide the comprehensive structural information of complicated compounds. To date, no report on evaluating the nutritional values of habitat soils has been presented for the host *Hepialus* larva of *O. sinensis* at the molecular level.

**Figure 1:**
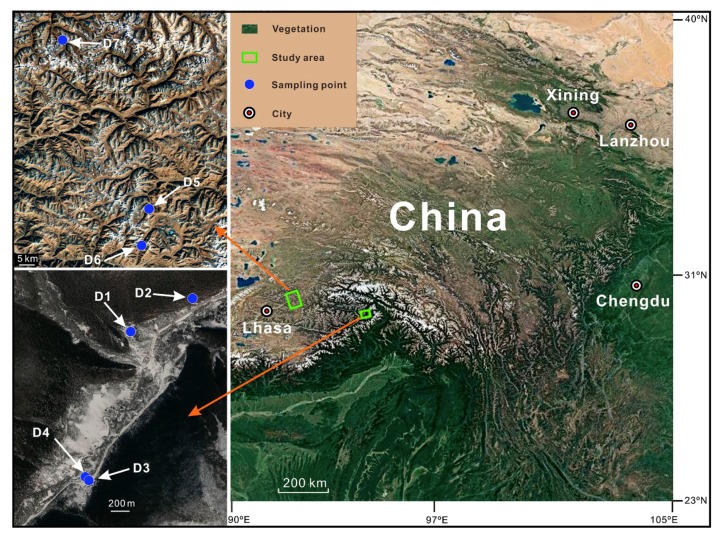
Schematic diagram illustrating the habitats of wild *Ophiocordyceps sinensis* in China and sampling localities in this study.

It is well known that lipid is one of the most important materials for *Thitarodes* larvae to protect against the cold in winter [7,34]. To adapt to the environment of low temperatures, typically at average annual temperatures below 5 °C, even cold temperatures as low as −12 °C, *Thitarodes* larvae may simultaneously accumulate lipids to maintain their cell functions [2,3,7,8]. In this paper, combining with the measured physico-chemical parameters of habitat soils and stable carbon isotope composition of major plant tender roots in the Sejila Mountain (Figure 1), we firstly characterize the structures of humic fractions and assess their nutritional value by FTIR and ^13^C NMR, and then discuss the possibility of humic factions as an alternative food source of *Thitarodes* larvae. Our results may further provide a technical support to establish artificial high-altitude habitats for reproducing *Thitarodes* larvae on the large scale with low cost, and further relax the contradiction between the sustainable utilization of *O. sinensis* resource and protection of vulnerable environments in the Qinghai-Tibetan Plateau.

## 2. Results

### 2.1. Physico-Chemical Properties of Habitat Soils

The pH values of habitat soils (Table 1) at the studied sites are presented in Table 2. It can be seen that their pH values range from 5.01 to 6.00 with a mean of 5.48, meaning acidic soils and no significant difference among different habitats. The pH values gradually increase with increasing altitude, but their variation among different soil layers in the same place is small.

**Figure 2:**
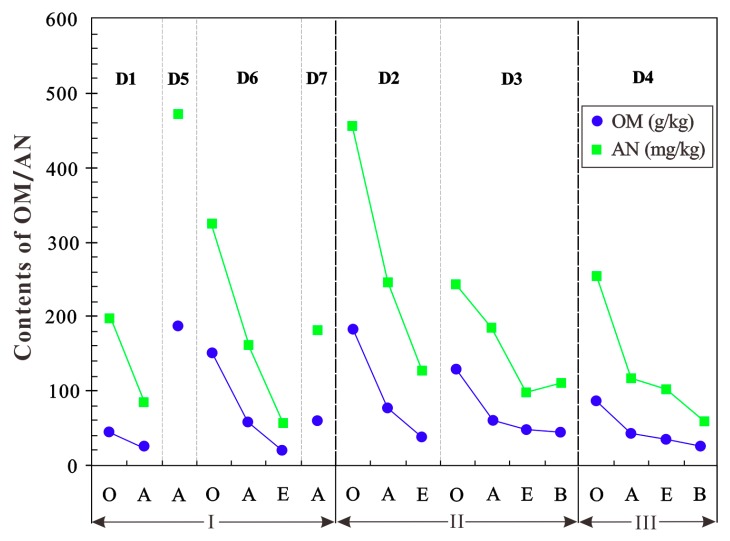
Variations of organic matters (OM) and available nitrogen (AN) in habitat soils in the Sejila Mountain, Tibet.

The contents of organic matters vary significantly from 20.7 g/kg to 187.2 g/kg, averaging 73.3 g/kg (Table 2). In the same point, the contents of organic matters reduce successively from layers O to A, to E, and to B, (Figure 2), especially reduction by more than half from layers O to A.

Similarly, the contents of total nitrogen are at the interval of 1.00–7.75 g/kg, with a mean of 3.14 g/kg; while the contents of available nitrogen also change greatly, from 55.1 mg/kg to 472.5 mg/kg with a mean of 193.3 mg/kg (Table 2). It can be seen from Figure 2 that there is a positive correlation between the contents of available nitrogen and organic matters, with the high correlation coefficients of 1.000 (D1), 0.985 (D6), 0.992 (D2), 0.827 (D3), and 0.995 (D4), respectively, and their associated *p* values are 0.20 (D1), 0.06 (D6), 0.17 (D2), 0.07 (D3), and 0.10 (D4), respectively. It is also clear that the contents of available nitrogen decrease sharply from layers O to A, and have a variation trend similar to that of organic matters. The contents of available nitrogen account for 4.08–7.78% of total nitrogen with a mean of 6.59% (Table 2).

As a whole, the physical and chemical parameters of the soils in three kinds of typical habitats in the Sejila Mountain are mutually overlapped, and generally have no evident differences.

### 2.2. Humic Fractions of Habitat Soils

**Figure 3:**
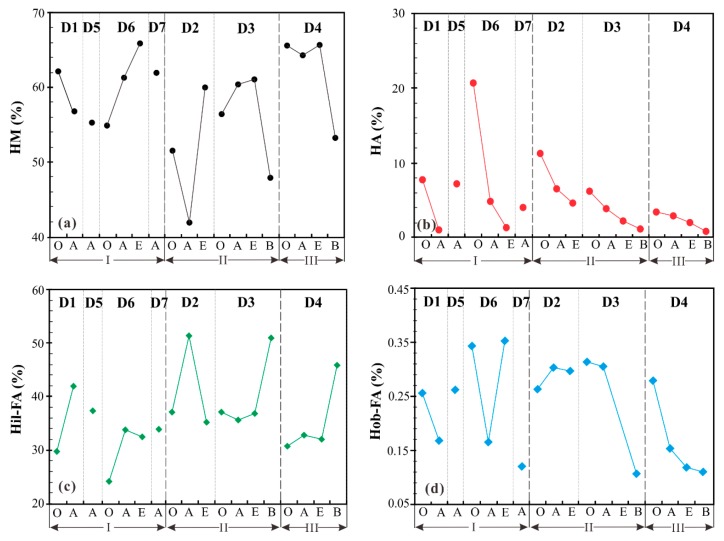
Variations of humic fractions from habitat soils in the Sejila Mountain, Tibet. (**a**): HM; (**b**): HA; (**c**): Hil-FA; (**d**): Hob-FA.

Based on the different solubility in acidic and alkaline solutions, humic substances can be divided into four fractions, which are humic acid (HA), hydrophilic fulvic acid (Hil-FA), hydrophobic fulvic acid (Hob-FA), and humin (HM) [12]. It can be seen in Table 3 that the percentages of fraction HM in all sites are the highest, ranging from 41.9% to 65.8%, and more than 50% in most cases. The other three fractions decrease in the order of Hil-FA (24.2–51.3%), HA (0.82–20.5%), and Hob-FA (0.11–0.35%). The vertical variations of fractions HA, HM, Hil-FA, and Hob-FA are illustrated in Figure 3 for different profiles of habitat soils. Variations of the same fraction in different points are quite different. The contents of fraction HA decrease in the order from layers O to A, to E, and to B; while the contents of fraction HM have no obvious variation trend. It is interesting that fractions HA and Hob-FA from the soils (except D4) decrease sharply for the habitats with the simultaneous occurrence of *Thitarodes* larvae and *O. sinensis*. However, the contents of fraction Hil-FA from the soils (except D3) evidently increase from layers O to A for the habitats with the appearance of *Thitarodes* larvae but no *O. sinensis*.

### 2.3. δ^13^C Values of Major Tender Plant Roots

Similar plants occur in the three habitats, mainly Polygonaceae, Rosaceae, Ranunculaceae, Ammonitaceae, Gramineaceae, and Cruciferous. The stable carbon isotope ratios for the tender roots of 22 plant species are presented in Table 4. It can be seen from Table 4 that their δ^13^C values range from −26.3‰ to −29.6‰, implying that they belong to C3 plants.

### 2.4. FTIR and ^13^C NMR Spectra

**Figure 4:**
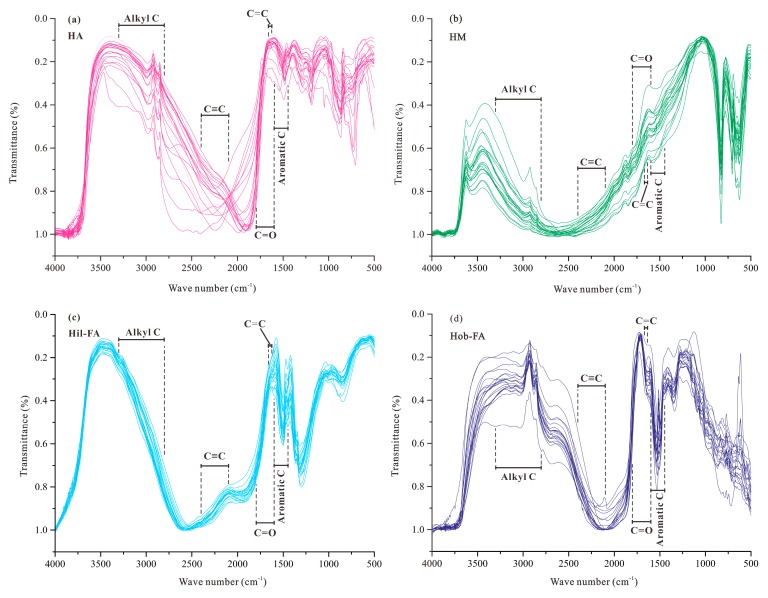
Fourier transform infrared spectroscopy (FTIR) spectra of humic fractions from habitat soils in the Sejila Mountain, Tibet. (**a**): HA; (**b**): HM; (**c**): Hil-FA; (**d**): Hob-FA.

**Figure 5:**
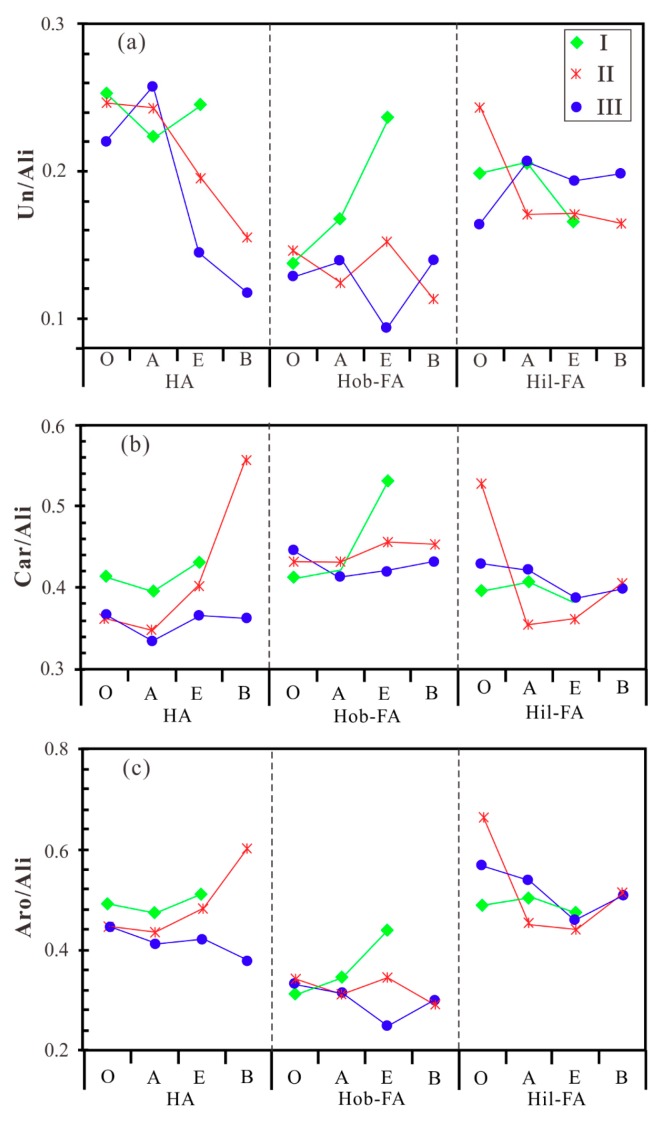
Variation of the ratios between unsaturated hydrocarbon and aliphatic carbon (Un/Ali) (**a**), carbonyl and aliphatic carbon (Car/Ali) (**b**), and aromatic carbon and aliphatic carbon (Aro/Ali) (**c**) in humic fractions from habitat soils on the basis of FTIR spectra. I, II, and III represent different habitat types.

The FTIR spectra of humic fractions in different habitat soils are illustrated in Figure 4. It can be seen that all of humic fractions split into two absorption areas with the wave numbers of 2000–2500 cm^−1^. The peaks are assigned to their corresponding functional groups by reference to the literature [31,35,36]. The peaks from the boundary to 4000 cm^−1^ are attributed to nitrogen or oxygen substituted alkanes, and aliphatic or unsaturated hydrocarbons; while the peaks from the boundary to 500 cm^−1^ denote carbonyl and benzene ring. Here, we integrate the peaks according to the following assignments, that is, the peak of 3300–2800 cm^−1^ denotes aliphatic carbon (Alkyl C); the peaks of 2400–2100 cm^−1^ and 1670–1640 cm^−1^ correspond to unsaturated hydrocarbons (C≡C and C=C); the peaks of 1800–1600 cm^−1^ and 1600–1450 cm^−1^ are attributed to carbonyl (C=O) and aromatic carbon (Aromatic C), respectively (Figure 4). Therefore, several parameters for functional groups in humic fractions can be defined as the ratios of integrated peak areas (Table 5), and their ratio variations for different habitat types are shown in Figure 5. The ratio between unsaturated hydrocarbon and aliphatic carbon (Un/Ali) in fraction HA is the highest, generally higher than 0.20; while the ratios in fractions Hob-FA and Hil-FA are mainly less than 0.20. Fraction Hob-FA has the highest average ratio (0.43) between carbonyl and aliphatic carbon (Car/Ali), while fraction HA possesses the lowest ratio (0.37). The ratio between aromatic carbon and aliphatic carbon (Aro/Ali) in fraction Hil-FA is the highest, mainly higher than 0.50; while the ratios for fraction Hob-FA range from 0.31 to 0.36. Overall, there is no significant difference between the three ratios for all fractions in different habitat types (Figure 5).

**Figure 6:**
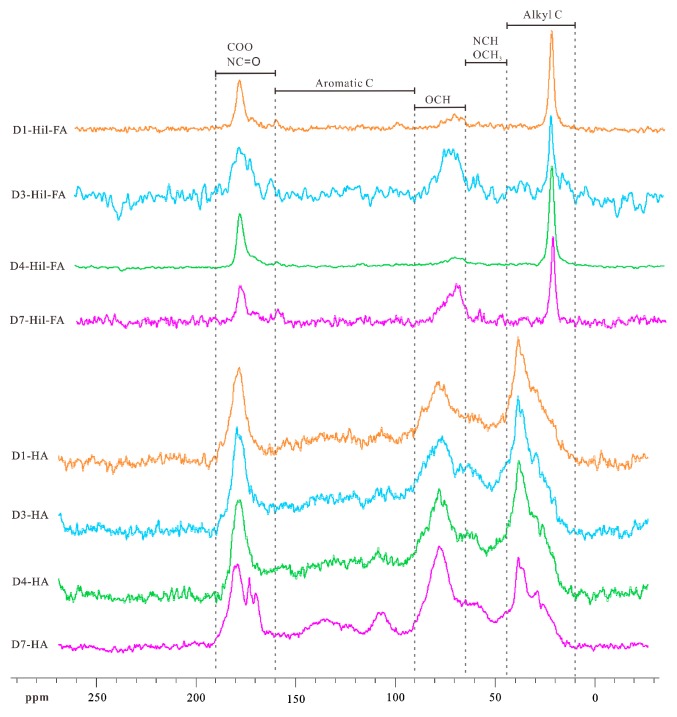
^13^C NMR spectra of fractions HA and Hil-FA from layer A in habitat soils in the Sejila Mountain, Tibet.

The solid-state ^13^C NMR spectra of fractions HA and Hil-FA are shown in Figure 6. Due to the extremely low contents of fraction Hob-FA, its ^13^C NMR data are unavailable. The peak area of fraction HA is concentrated in the following four regions: 186–160 ppm, 90–64 ppm, 64–44 ppm, and 44–10 ppm. The ^13^C NMR signals are assigned to the corresponding functional groups according to previous studies [35,37,38,39]. For fraction HA, two peaks can be recognized in the region of 10–64 ppm, that is, 10–44 ppm for aliphatic carbon (Alkyl C), and 44–64 ppm for NCH or OCH_3_. In the region of 64–160 ppm, two peaks represent carbohydrate OCH (64–90 ppm) and aromatic carbon (Aromatic C) (90–160 ppm), respectively. For the C=O region, COO/NC=O exhibits in the spectrum (160–180 ppm). For fraction Hil-FA, its peak area is concentrated in the following three regions: 190–160 ppm, 90–64 ppm, and 30–20 ppm. In the aliphatic region, group CH_2_ exhibits in the spectrum (20–30 ppm). In the carbohydrate region, group OCH appears in the spectrum (64–90 ppm). In the C=O region, the functional groups of COO and NC=O display in the spectrum (160–190 ppm).

Based on the integral areas of specific peaks, we calculated the parameters for functional groups, as shown in Table 6. For fraction HA, the ratios of (NCH or OCH_3_)/aliphatic carbon range from 0.32 to 0.47 with a mean of 0.41; the ratios between aromatic carbon and aliphatic carbon range from 0.64 to 0.96 with a mean of 0.74. It is notable that these two ratios are unavailable for fraction Hil-FA owing to its too small peak area. For fraction HA, the ratios between carbohydrate OCH and aliphatic carbon (0.69–1.01, with a mean of 0.79) and the ratios between COO/NC=O and aliphatic carbon (0.32–0.79, with a mean of 0.50) have the slight differences among three types of habitats. However, these two ratios differ greatly in fraction Hil-FA, in particular between carbohydrate OCH and aliphatic carbon. It can be seen in Table 6 that the ratios between carbohydrate OCH and aliphatic carbon in fraction Hil-FA range from 0.30 to 1.48 with a mean of 0.97; and the ratios between COO/NC=O and aliphatic carbon range from 0.83 to 1.47 with a mean of 1.10. These two ratios for fraction Hil-FA are generally higher than those of fraction HA. The number of COO/NC=O is smaller in that of fraction HA, which is consistent with that of the FTIR signal. It is interesting that the ^13^C NMR data reveal the existence of more aromatic functional groups in fractions HA than in Hil-FA; while the FTIR data reflect no apparent difference in functional groups between fractions HA and Hil-FA. On the whole, the abovementioned ratios indicate that fraction HA has more aliphatic functional groups than the other humic fractions.

## 3. Discussion

### 3.1. Nutrition Assessment of Humic Substances from Habitat Soils

Many investigators have discussed the relationship between the body fat of insect larvae and their cold tolerance [40,41,42,43,44]. It is consensus that for organisms under cold stress, lipid is the most crucial limiting nutrient. It is well known that *Thitarodes* larvae annually experience winter cold temperatures as low as −12 °C [11,34,44,45]. We had observed that the larvae did not have any feeding behavior in the extremely chilling days when they became frozen stiff. Thus, they preferred to store abundant fats before being frozen, and their fat contents accordingly increased significantly in the early winter [44,46]. The formers demonstrated that *Thitarodes* larvae in the Sejila Mountain had the fat contents of 17.10–21.87%, apparently higher than those in low-altitude areas [7,44]. Because the uptake of molecular segments for biosynthesizing nutrients from the food source directly is energetically more efficient, especially in the cold winter [47], whether or not humic substances enrich molecular segments for biosynthesizing lipids is an important criterion for evaluating their nutritional value.

Generally, humic fractions are structurally similar, but evidently differ in their molecular weights and functional groups [48]. Although the formers have made extensive efforts, there is no consensus on the exact composition and structure of humic fractions. In this study, FTIR and ^13^C NMR analyses demonstrate that humic fractions from habitat soils contain plentiful functional groups, which may be utilized by *Thitarodes* larvae for synthesizing essential nutrients, lipids in particular. It is well-established that the quality of each food depends on how well its nutrients made available by digestion fit the nutritional requirements of an insect [49]. If the nutritional qualities of humic fractions from soils are indeed different, one would expect a higher metabolic efficiency in *Thitarodes* larvae fed with the humic fraction with higher nutritional quality (higher contents of proteins, carbohydrates, lipids, and other nutrients) [50]. Although having complicated structures, humic substances mainly include aliphatic and aromatic moieties [51]. In terms of the efficiency of biosynthesizing lipids, fraction HA has the highest nutritional value; fraction Hob-FA is higher than fractions HM and Hil-FA; and fraction HM may be the lowest, because fractions HA and Hob-FA have lipid groups more plentiful than the others [49,50,52]. It is notable that dietary lipids, particularly essential fatty acids, are critical nutritional components, because they cannot be synthesized in sufficient qualities by animals [53], and thus have to be ingested via diet. These results imply that fractions HA and Hob-FA in the study area may be a high-quality potential food for *Thitarodes* larvae. From the standpoints of compositions and structures, the humus-rich horizon in habitat soils, that is, layer A enriches molecular segments for biosynthesizing nutrients by *Thitarodes* larvae.

### 3.2. Humus-Rich Horizon to Be a Suitable Feeding Locality for Thitarodes Larvae

The properties of habitat soils usually control the distribution, abundance, and activity of the biota [54,55,56]. For instance, the pH value is known to have a significant influence on the activity of the biota in a habitat soil, such as affecting the chemical forms of humic substances, as well as concentrations and availability of nutrients [57]. Organic matters and nitrogen in soils are two important components, which have diverse beneficial effects on the statuses and structures of their nutrients, and thus control the biota activity [54,58]. As we know, *Thitarodes* larvae usually live in the humus-rich horizon of soils, that is, layer A, because humic substances in layer A contain a large amount of nutrients for organisms [56]. Some cave animals, such as earthworms, may live in more extreme environments with the pH interval of 4.3–9.2, showing the stronger adaptability to environments [59]. Previous observations also revealed that the slightly acidic condition was well suitable for the growth of *O. sinensis* mycelia [11,45]. Based on the features of physico-chemical parameters of habitat soils in this study, it can be inferred that layer A may be the suitable locality for the survival and feeding of *Thitarodes* larvae.

It is noteworthy that there is no evident difference in the molecular structures of humic fractions as well as physico-chemical properties of soils among three typical kinds of habitats in the Sejila Mountain. Because no replicates have been performed for each sampling site in this study, further studies are needed to confirm the universal features of the molecular structures of humic fractions and physico-chemical parameters in these typical habitat soils in the Sejila Mountain.

### 3.3. Humic Substances to Be an Alternative Food Source of Thitarodes Larva

Generally, the tender roots of plants such as Polygonaceae and Cuculidae, particularly *Polygonum macrophyllum* and *Polygonum viviparnm*, are the favorite foods of *Thitarodes* larvae [12]. Carrot, yam and potato are also their favorite foods in artificial feeding [60]. However, plants in the high-altitude habitat of *O. sinensis* have a very short growth period (only in summer). Thus, feeding on humic substances in habitat soils during the cold winter (mainly from November to April) becomes an unavoidable demand for *Thitarodes* larvae in the absence of tender plant roots.

Geophagy is a common phenomenon for faunas in soils, such as earthworms, termites, and ants, mainly for their nutritional demand [51,61,62]. Earthworm is considered to be an ecosystem engineer [59,63,64,65,66], who deeply modifies the physical, chemical and biological properties of soils and their functions via feeding, burrowing and casting [62,67]. Direct and indirect evidence has suggested that earthworms can digest the complicated organic compounds fairly well [62]. Feeding termites with soils has been also investigated, elucidating the digestion mechanism of humic substances from soils [61]. Another benefit of humic substances, especially fraction HA is able to provide diverse micronutrients for faunas in soils [21,26,68,69,70,71,72]. Similarly, *Thitarodes* larvae may also obtain diverse nutrients, including micronutrients from their habitat soils.

With regard to the diet of *Thitarodes* larvae, Zhu and He [18] speculated that humic substances in habitat soils might be their food source, but no evidence was provided. It is demonstrated that stable carbon isotope analysis has a remarkable advantage in tracing the long-term diet of animals [73]. Adopting stable carbon isotope analysis, our group ever investigated the diet of *Thitarodes* larvae for the first time, indicating that there were two types of *Thitarodes* larvae; one with the δ^13^C values of −22.6‰ to −23.4‰ chiefly fed on soil humic substancs, and the other with the δ^13^C values of −24.6‰ to −27.6‰ mainly ate tender plant roots [12]. Further, a digital calculation was performed on the basis of the stable carbon isotope composition, revealing that fraction HA in the Sejila Mountain was utilized by larvae up to 66.7–78.3% in the cold winter. In this study, the compositions and structures of humic substances, which were mainly derived from C3 plants, indicate that fractions HA, Hob-FA, and Hil-FA contain abundant molecular segments for biosynthesizing nutrients, and accordingly may be an ideal food source for *Thitarodes* larvae in the absence of tender plant roots. It should be pointed out that there is a transformation from fractions HA to FA in the guts of geophagous earthworms via the ^14^C labeling method [51], meaning that the activities of the faunas in soils can change the proportion of humic fractions. Thus, any simple claim on the food source of *Thitarodes* larvae inferred only from the composition of humic fractions and their changes may be misleading.

Many studies have demonstrated that the host *Thitarodes* larvae of *O. sinensis* have a hunger-resistance ability, and fat storage is one way to fight hunger. For instance, Chen et al. [74] found that *Thitarodes* larvae could live in habitat soils without the addition of any plant food for a long time. *Thitarodes* larvae might also live for a relatively short term (up to 119 days) without habitat soils [74,75]. Thus, the host *Thitarodes* larvae not only possess strong hunger-resistance, but also have the ability to utilize the nutrients in habitat soils as their food, which is consistent with our previous [12] and newly-obtained results. To verify the possibility of humic substances from habitat soils as an alternative food source of *Thitarodes* larvae, an artificially breeding experiment in the high-altitude laboratory in the Sejila Mountain was performed by our group [76], revealing that they could live and grow fairly well in habitat soils without any supplement of tender plant roots or tubers for at least half a year. Thus, the current available evidence strongly supports humic substances in habitat soils as an optional food source for *Thitarodes* larvae.

*O. sinensis* has been widely studied for its mysterious life history, but many challenges, especially how to commercially produce this glorified herbal medicine, are still hard to fully overcome. Meanwhile, these challenges also may induce some derivative issues, such as product authenticity, quality, and safety [11]. Therefore, our newly-obtained results may provide a novel idea for performing an eco-friendly and semi-wild cultivation of *Thitarodes* larvae with low cost, and relieving the contradiction between the sustainable utilization of *O. sinensis* resource and protection of vulnerable environments in the Qinghai-Tibetan Plateau.

## 4. Materials and Methods

### 4.1. Sampling and Pretreatment

Based on habitat status of whether *Thitarodes* larvae and *O. sinensis* occur or not, the soil samples were collected from the humus layer in the Sejila Mountain on the Tibetan Plateau on 20 June 2007 (Figure 1). Seven sampling points were located in Nyingchi, Maizhokunggar, and Damxung, as shown in detail in Table 1. From the surface downwards, the soils at the sampling points may be divided into four layers, which are the litter layer (layer O, 7–9 cm), humus layer (layer A, 23–33 cm), leached layer (layer E), and accumulation layer (layer B). The sampling point is located at the shrubbery, and the main bush is *Rhododendron nivale*, whose roots extended as deep as 45 cm. The soil samples were collected in different layers at each sampling point according to the zonation of a soil profile. The tender roots of major plants in this area were accordingly sampled and photographed with a high-resolution digital camera for indoor identification.

The dried soil samples were directly ground into the powders with a size of less than 100 meshes, and then were separated into humic acid (HA), hydrophilic fulvic acid (Hil-FA), hydrophobic fulvic acid (Hob-FA), and humin (HM) by organic chemistry [12]. In brief, about 20 g of soil powders were weighed, and then 200 mL of 0.1 mol/L NaOH and 5 mL of 3% NaCl were added; input N_2_, stirred and stood overnight at room temperature; finally centrifuged the mixed solution and collected the supernatant (HA + FA). The indissoluble part was humin (HM) after the removal of pyrite and silicates by thick HCl and HF. Acidified the supernatant (HA + FA) to pH = 1.2 with 4 mol/L HCl, stood overnight; centrifuged and obtained the precipitate of HA; the filtrate was FA after passing the filter membrane with an aperture of 0.22 μm. Added 100 mL ethyl acetate into FA, and then separated the hydrophobic layer (containing hydrophobic fulvic acid, Hob-FA) and hydrophilic layer (containing hydrophilic fulvic acid, Hil-FA) by a separatory funnel; evaporated the ethyl acetate layer to get Hob-FA; added 0.1 mol/L AlCl_3_ and 1 mol/L NaOH to the hydrophilic layer (Hil-FA), made the solution to pH = 5.0, stood overnight, and then obtained Hil-FA by centrifugation. Added the mixed solution of 0.3 mol/L HF and 0.1 mol HClO_4_ into HA, continuously stirred for 5 h at room temperature to remove fine minerals, then obtained a precipitate (HA) by centrifugation; entirely re-dissolved HA with a 0.1 mol NaOH solution, then dialyzed until the electrical conductivity of the outer equilibrated solution less than 10 μS/cm, and finally obtained HA after the solution in the dialysis bag was evaporated.

All separated fractions were dried and manually ground to fine-grained powders before the following analysis. Tender plant roots were dried in the oven at 40 °C, and then milled into 40 mesh powders by a glass mortar before analysis.

### 4.2. Analytical Methods

The pH values in habitat soils were determined in the field by the glass electrode method (soil-to-water ratio, 1:1) [77]. The contents of organic matters (organic carbon) were detected by the potassium dichromate volumetric method [78]. The contents of total nitrogen and available nitrogen were measured by the Kjeldahl method [79] and alkali diffusion method, respectively [80]. The δ^13^C values of tender plant roots were measured by a CE EA1112 C/N elemental analyzer (CE Instruments, Wigan, UK)-Delta^plus^ XL stable isotope ratio mass spectrometry (Finnigan, Thermo Scientific, Waltham, MA, USA). The analytical errors were less than 0.3‰ for δ^13^C values. The analytical procedures in detail were given by Liu et al. [81].

The Fourier transform infrared spectra (FTIR) of humic fractions were generated via an EQUINOX 55 Fourier spectrometer (Bruker Optics, Ettlingen, Germany). The measuring region ranged from 500 cm^−1^ to 4000 cm^−1^. The KBr pellet was prepared by pulverizing approximately 2 mg of each dried humic fraction with 100 mg of KBr powder in an agate mortar, and then the mixture was pressed into a pellet under the pressure of 1400 kg/cm^2^.

The solid-state ^13^C NMR CPTOSS measurements were performed on a Bruker Avance 400 (400 MHz) spectrometer (Bruker Bio-Spin, Zurich, Switzerland) with an MAS rate of 6 kHz. The adequate amount of each powder sample was loaded, and the chemical shifts were at the interval of 20–200 ppm. The ^13^C NMR spectra were recorded with about 6000–7500 scans, 3.0 s relaxation delay (D1) between adjacent scans, 50 ms acquisition time. An APT experiment from the Bruker library was used with 1 k accumulated scans, and the plot limits ranged from +260 ppm to −20 ppm. The total signal intensity and proportion contributed by each functional group were determined by integrating the spectra of their chemical-shift areas. The relative contents of different functional groups were calculated from the areas on the basis of their ^13^C NMR spectra, and expressed as the percentages of the areas to aliphatic carbon spectra.

### 4.3. Statistical Analysis

The experimental data were analyzed by using the IBM SPSS Statistics (Ver. 20, Microsoft, Chicago, IL, USA). The δ^13^C value for each sample of tender plant roots was determined three times. Their standard deviations are less than 0.3‰, and are expressed as the mean values (Table 4).

## 5. Conclusions

Humic substances in habitat soils in the Sejila Mountain, Tibet contain abundant molecular segments for synthesizing essential nutrients, especially lipids produced by *Thitarodes* larvae. FTIR and ^13^C NMR analyses suggest that fraction HA may possess the superior supply of raw materials for biosynthesizing lipids to other fractions. Both fractions Hob-FA and Hil-FA have the potential supply of raw materials, but fraction Hob-FA is better. The possibility of humic factions as an alternative food source of *Thitarodes* larvae is further confirmed by the previous artificially-bred *Thitarodes* larvae experiment and stable carbon isotope evidence. Overall, humic substances mainly originating from C3 plants in habitat soils in Tibet have the ability to provide the vital nutrients, and thus may be an important food source for *Thitarodes* larvae, especially in the cold winter.

## Figures and Tables

**Table 1:** Information of sampling points in three habitats in the Sejila Mountain, Tibet.

Samples	Longitude	Latitude	Location	Altitude (m)	Notes
D1	94°36′16 E	29°36′20 N	Nyingchi	4200	Both *Thitarodes* larva and *O. sinensis* occurred (13 larvae/m^3^).
D2	94°36′32 E	29°36′29 N	Nyingchi	4242	*Thitarodes* larva but no *O. sinensis* occurred (2 larvae/m^3^).
D3	94°36′03 E	29°35′36 N	Nyingchi	4241	*Thitarodes* larva but no *O. sinensis* occurred (40 larvae/m^3^).
D4	94°36′03 E	29°35′37 N	Nyingchi	4137	Both *Thitarodes* larva and *O. sinensis* did not occur.
D5	92°19′53 E	29°50′21 N	Maizhokunggar	4825	Both *Thitarodes* larva and *O. sinensis* occurred (1 larva/m^2^).
D6	92°18′46 E	29°44′50 N	Maizhokunggar	4820	Both *Thitarodes* larva and *O. sinensis* occurred (3 larvae/m^2^).
D7	92°06′29 E	30°16′37 N	Damxung	4805	Both *Thitarodes* larva and *O. sinensis* occurred (1 larva /m^2^).

**Table 2:** Physical and chemical parameters of habitat soils in the Sejila Mountain, Tibet.

Habitat Types	Samples	Layers	pH	OM (g/kg)	TN (g/kg)	AN (mg/kg)	AN/TN
I: both *Thitarodes* larva and *O. sinensis* occurred.	D1	O	5.01	44.3	2.73	196.9	7.21
		A	5.13	25.0	1.26	85.1	6.76
	D5	A	6.00	187.2	7.75	472.5	6.10
	D6	O	5.60	150.8	5.82	324.4	5.57
		A	5.85	58.1	2.64	160.7	6.09
		E	5.88	20. 7	1.10	55.1	5.02
	D7	A	5.91	59.9	3.19	181.1	5.69
II: *Thitarodes* larva but no *O. sinensis* occurred.	D2	O	5.55	183.7	6.99	456.8	6.54
		A	5.46	79.4	3.35	247.3	7.38
		E	5.09	37.6	1.85	126.8	6.87
	D3	O	5.49	129.5	5.98	244.1	4.08
		A	5.24	60.1	2.63	183.9	7.00
		E	5.32	47.7	2.03	99.2	4.89
		B	5.40	44.4	1.91	110.3	5.79
III: both *Thitarodes* larva and *O. sinensis* did not occur.	D4	O	5.62	87.2	3.28	255.2	7.78
		A	5.45	43.3	1.76	118.1	6.73
		E	5.29	34.6	1.33	102.4	7.71
		B	5.38	25.3	1.00	59.9	5.97

Notes: Organic matters, OM; TN, Total nitrogen; and AN, Available nitrogen.

**Table 3:** Contents of humic fractions from the habitat soils in the Sejila Mountain, Tibet.

Layers	Samples	HM (%)	HA (%)	Hil-FA (%)	Hob-FA (%)
O	D1	62.1	7.84	29.8	0.26
	D2	51.4	11.3	37.1	0.26
	D3	56.5	6.18	37.0	0.31
	D4	65.7	3.38	30.7	0.28
	D6	54.9	20.5	24.2	0.34
A	D1	56.7	1.07	42.0	0.17
	D2	41.9	6.45	51.3	0.30
	D3	60.4	3.75	35.5	0.30
	D4	64.3	2.87	32.7	0.15
	D5	55.3	7.09	37.4	0.26
	D6	61.3	4.68	33.8	0.17
	D7	62.0	3.95	33.9	0.12
E	D2	60.0	4.51	35.2	0.30
	D3	61.1	2.12	36.8	
	D4	65.7	1.92	32.3	0.12
	D6	65.8	1.2	32.6	0.35
B	D3	48.0	1.08	50.8	0.11
	D4	53.3	0.82	45.8	0.11

**Table 4:** Average δ^13^C values of major tender plant roots in the Sejila Mountain, Tibet.

Plant Species	δ^13^C (‰)	Plant Species	δ^13^C (‰)
*Aster barbellatus*	−27.6	*Fragaria nubicola*	−28.7
*Aster souliei*	−28.0	*Geranium melananthum*	−26.4
*Aconitum longilobum*	−28.5	*Gremanthodium thomsonii*	−26.3
*Aletris stelliflora*	−26.3	*Pedicularis mollis*	−26.4
*Caltha scaposa*	−28.8	*Polygonum viviparum*	−27.8
*Conringia planisiliqua*	−29.0	*Potentilla stenophylla*	−27.2
*Cremanthodium lingulatum*	−26.9	*Ranunculus tanguticus*	−26.7
*Delphinium kamaonense*	−26.4	*Rhododendron nivale*	−27.6
*Deyeuxia nivicola*	−29.4	*Rumex nepalensis*	−26.8
*Deyeuxia nyingchiensis*	−29.4	*Saussurea sungpanensis*	−27.6
*Eryaimum hieracifolium*	−29.6	*Selaginella vardei*	−27.8

**Table 5:** Structure parameters derived from the FTIR spectra of humic fractions from the habitat soils in the Sejila Mountain, Tibet.

Fractions	Samples	Un/Ali	Car/Ali	Aro/Ali
HA	D1	0.13	0.50	0.56
	D5	0.29	0.38	0.45
	D6	0.25	0.33	0.45
	D7	0.22	0.37	0.45
	D2	0.24	0.37	0.45
	D3	0.25	0.33	0.42
	D4	0.26	0.33	0.41
Hob-FA	D1	0.22	0.44	0.36
	D5	0.12	0.41	0.34
	D6	0.19	0.41	0.36
	D7	0.14	0.45	0.34
	D2	0.13	0.43	0.32
	D3	0.12	0.43	0.31
	D4	0.14	0.41	0.32
Hil-FA	D1	0.20	0.39	0.50
	D5	0.23	0.46	0.59
	D6	0.20	0.42	0.52
	D7	0.19	0.36	0.42
	D2	0.18	0.38	0.50
	D3	0.16	0.33	0.41
	D4	0.21	0.42	0.54

**Notes**: Un/Ali represents the ratio between unsaturated hydrocarbon and aliphatic carbon; Car/Ali represents the ratio between carbonyl and aliphatic carbon; and Aro/Ali represents the ratio between aromatic carbon and aliphatic carbon.

**Table 6:** Structure parameters derived from the ^13^C nuclear magnetic resonance spectrum (^13^C NMR) spectra of fractions humic acid (HA) and hydrophilic fulvic acid (Hil-FA) from layer A in habitat soils in the Sejila Mountain, Tibet.

Samples	Aliphatic Carbon	NCH/OCH_3_/Aliphatic Carbon	Carbohydrate OCH/Aliphatic Carbon	Aromatic Carbon/Aliphatic Carbon	COO/NC=O/Aliphatic Carbon
D1-HA	1	0.32	0.69	0.64	0.44
D3-HA	1	0.44	0.76	0.66	0.43
D4-HA	1	0.40	0.71	0.68	0.32
D7-HA	1	0.47	1.01	0.96	0.79
D1-Hil-FA	1	−	0.54	−	1.08
D3-Hil-FA	1	−	1.55	−	1.47
D4-Hil-FA	1	−	0.30	−	0.83
D7-Hil-FA	1	−	1.48	−	1.04

Note: “−” means no detected.
